# Inebriety and the Legislature

**Published:** 1892-02-13

**Authors:** 


### INEBREITY AND THE LEGISLATURE.

The Society for the Study of Inebriety is but one of the many evidences that the medical profession are taking the profoundest interest in the social problems of the day. If it can be said of some of the sister professions that they are led and guided by public opinion, where they ought to be leading and directing it, such an accusation cannot be brought against the medical faculty at the present time, although in the past they may have failed to take the active part in the repression of inebriety that we may justly expect from a profession who, as a body, have devoted themselves to improving the physical welfare of humanity at large.

From a recent number of the "Proceedings" of this Society we learn much that is interesting and worthy of note. Chevalier Max de Proskowetz contributes a paper on the punishment of inebriety itself, and the responsibility of drunkards for a crime they committed when drunk, according to Austrian law.

Although simple inebriety is not yet punishable in Austria, we are glad to see that a Bill was proposed in June by the Minister to the Austrian Parliament, which is called Law for Checking Inebrity, and is valid for all Austria. One section of this Bill enacts that whoever is evidently drunk in inns or liquor shops, in rooms where sellings, or retail, or delivering of liquors takes place, or in the streets, or at any other public place, and whoever at such places makes another drunk is punished by prison or fine. The same punishment rightly applies to innkeepers who supply the liquor.

It certainly seems absurd not to make the fact of being drunk punishable, when it is the fruitful source, we may say the immediate cause, of so many unpremeditated crimes being committed in an irresponsible state. In England we do not allow that a man being drunk, and, as a matter of fact, irresponsible, diminishes his legal responsibility, and yet we do not punish a man for putting himself into an irresponsible condition.*

During the discussion of the Chevalier's paper, the President, Dr. Norman Kerr, called attention to the remarkable difference between Austrian and British jurisprudence as regards inebriate criminal responsibility. "In England, a man having while intoxicated killed his friend, would probably have been hung or sentenced to imprisonment for life, or for a long period. By this procedure he would either have been put out of existence with little chance for repentance, or he would probably have become a hopeless and irreclaimable criminal. By the juster Austrian penal code, Lagodin, who had killed his best friend in the unconscious frenzy of drunkenness, had been sentenced to only one and a half years' imprisonment, on the ground that there had been no criminal intention. By the latter procedure Lagodin had the opportunity of turning over a new leaf, had availed himself of this opportunity, and had after his release become a good citizen, a trustworthy, a well-conducted man, a public benefactor, and leader of a determined and successful attempt to deal with the prevailing inebriety of his fellow-countrymen. Britain might well profit by such a happy illustration of a discriminating distinction in punishment between crimes done in intentional and unintentional intoxication."

The journal also contains the report of the Inebriates' Legislation Committee of the British Medical Association, and other articles of interest.

* The Emperor of Germany is at present endeavouring to legislate on this very difficult question. It is by no means a simple matter to deal with practically, but we may sincerely hope that a workable measure may be devised, and his brave attempts to cope with existing social evils may be crowned with success.

